# HIV-tat alters Connexin43 expression and trafficking in human astrocytes: role in NeuroAIDS

**DOI:** 10.1186/s12974-016-0510-1

**Published:** 2016-03-02

**Authors:** Joan W. Berman, Loreto Carvallo, Clarisa M. Buckner, Aimée Luers, Lisa Prevedel, Michael V. Bennett, Eliseo A. Eugenin

**Affiliations:** Department of Pathology, Albert Einstein College of Medicine, Bronx, NY USA; Department of Microbiology and Immunology, Albert Einstein College of Medicine, Bronx, NY USA; Department of Neuroscience, Albert Einstein College of Medicine, Bronx, NY USA; Public Health Research Institute (PHRI), 225 Warren Street, Newark, NJ 07103 USA; Department of Microbiology and Molecular Genetics, Rutgers University, 225 Warren Street, Newark, NJ 07103 USA; Current address: Laboratory of Immunoregulation, NIAID, Bethesda, MD USA

**Keywords:** Gap junctions, AIDS, Dementia, Glia, Connexin

## Abstract

**Background:**

HIV-associated neurocognitive disorders (HAND) are a major complication in at least half of the infected population despite effective antiretroviral treatment and immune reconstitution. HIV-associated CNS damage is not correlated with active viral replication but instead is associated with mechanisms that regulate inflammation and neuronal compromise. Our data indicate that one of these mechanisms is mediated by gap junction channels and/or hemichannels. Normally, gap junction channels shutdown under inflammatory conditions, including viral diseases. However, HIV infection upregulates Connexin43 (Cx43) expression and maintains gap junctional communication by unknown mechanism(s).

**Methods:**

Human primary astrocytes were exposed to several HIV proteins as well as to HIV, and expression and function of Connexin43- and Connexin30-containing channels were determined by western blot, immunofluorescence, microinjection of a fluorescent tracer and chromatin immunoprecipitation (ChIP).

**Results:**

Here, we demonstrate that HIV infection increases Cx43 expression in vivo. HIV-tat, the transactivator of the virus, and no other HIV proteins tested, increases Cx43 expression and maintains functional gap junctional communication in human astrocytes. Cx43 upregulation is mediated by binding of the HIV-tat protein to the Cx43 promoter, but not to the Cx30 promoter, resulting in increased Cx43 messenger RNA (mRNA) and protein as well as gap junctional communication.

**Conclusions:**

We propose that HIV-tat contributes to the spread of intracellular toxic signals generated in a few HIV-infected cells into surrounding uninfected cells by upregulating gap junctional communication. In the current antiretroviral era, where HIV replication is often completely suppressed, viral factors such as HIV-tat are still produced and released from infected cells. Thus, blocking the effects of HIV-tat could result in new strategies to reduce the damaging consequences of HIV infection of the CNS.

## Background

HIV enters the CNS early after infection, and CNS viral persistence results in many neurological abnormalities [1, 2]. As the prevalence of HIV-associated neurocognitive disorders (HAND) increases [2, 3], understanding the mechanisms mediating the pathogenesis of HAND becomes even more critical. Currently, there is no correlation between the low to undetectable levels of HIV replication and extensive CNS damage. Therefore, several groups suggested that this damage is due to amplification systems used by the virus to increase HIV-associated inflammation. We propose that connexin (Cx)-containing gap junctions (GJ) and hemichannels (HC) are important in the amplification of this disease process.

Gap junctions are aggregates of channels connecting the cytoplasmic compartments of the coupled cells and provide direct continuity between the cells allowing electrical and metabolic coordination [4, 5]. A GJ channel is formed by the docking of two hemichannels (one contributed by each of the joined cells), and each hemichannel is composed of six protein subunits termed connexins (Cx). Recent evidence indicates that hemichannels composed of Cx in non-junctional membranes can open to the extracellular space under appropriate conditions and allow diffusional exchange between the cytoplasmic compartment and the extracellular environment, including highly inflammatory factors such as ATP, glutamate, and prostaglandins [4, 5]*.*

Astrocytes are the most abundant cell type in the CNS and are highly coupled by gap junctions containing Cx43 and Cx30 to coordinate metabolic and electric events [5], and only recently, there is increasing evidence of their significance in HIV infection. Despite their low viral replication and percentage of HIV-infected astrocytes present in vivo and in vitro (5 to 19 %), significant changes in astrocyte gene expression [6], apoptosis [7–10], glutamate metabolism, and blood brain barrier (BBB) [7, 8] have been reported, suggesting that these cells play a key role in NeuroAIDS.

Our laboratories demonstrated that despite low replication and numbers of HIV-infected astrocytes, Cx containing channels, including gap junctions and hemichannels, allow toxins from these few HIV-infected astrocytes to reach neighboring uninfected CNS cells [4, 7, 8, 11]. In our studies, it was surprising that connexin channels in human astrocytes are maintained or increased in response to HIV infection. Generally, Cx expression and gap junctional communication are decreased under inflammatory conditions, including viral infections [4, 12]. However, HIV infection of astrocytes results in opening of hemichannels, increased Cx43 expression, and maintenance of gap junctional communication [4, 7, 8, 11]. In this report, we demonstrate that expression of Cx43, but not of Cx30, is upregulated in HIV-infected conditions, especially in tissues obtained from HIV-infected individuals with cognitive impairment. In addition, we demonstrate that HIV-tat protein binds to the Cx43 promoter, resulting in an increase in Cx43 mRNA and protein expression as well as in the maintenance of gap junctional communication. Our results of the maintenance of Cx43 expression may explain how few HIV-infected astrocytes spread intracellular toxic signals into surrounding uninfected cells.

## Methods

### Materials

DMEM, fetal bovine serum (FBS), penicillin/streptomycin (P/S), and trypsin-EDTA were from Invitrogen (Grand Island, NY). Monoclonal antibody to GFAP, FITC or Cy3- conjugated anti-rabbit IgG, and Cy3 or FITC-coupled anti-mouse IgG antibodies were from Sigma (St. Louis, MO). Purified mouse IgG_2B_ and IgG_1_ myeloma proteins were from Cappel Pharmaceuticals, Inc. Lipofectamine 2000; anti-connexin43 antibody; anti-connexin30 antibody; and secondary antibodies anti-mouse, anti-rabbit, and anti-goat immunoglobulins conjugated to Alexa-533, Alexa-633, and Alexa-588 were from Life Technologies (Eugene, OR). Small interfering RNA (siRNA) for Cx30 and controls were from Santa Cruz Biotechnology (Santa Cruz, CA). HIV recombinant proteins, vif, nef, rev, gag (p55, p24, and p7), polymerase, and gp120 as well as HIV_ADA_ and HIV_JR-CSF_ were from the NIH repository (Germantown, MD). HIV-tat protein was purchased from the University of Kentucky or was a gift from Dr. Avindra Nath (NINDS, NIH, MD). We selected concentrations from 1 to 300 ng/ml due to the effects on neuronal toxicity (100 ng/ml induces neuronal apoptosis in 50–80 % of the cells and lower concentrations induce minimal apoptosis, 10–20 % [13, 14]). In addition, HIV-tat induced chemotaxis was also maximal at 100 ng/ml [15]. Concentrations below 100 ng/ml only induced minimal microglia migration. Thus, the concentrations selected (1 to 300 ng/ml) are appropriate in different models.

#### Brain tissue sections

Human brain tissue sections (10 to 20 μm) were obtained from the National NeuroAIDS Tissue Consortium (NNTC). This consortium is a NIH-funded (U24MH100925) source to explore the devastating consequences of HIV infection (www.nntc.org).

### Human astrocyte cultures and HIV infection

Cortical human fetal tissue was obtained as part of a research protocol approved by the Albert Einstein College of Medicine and Rutgers University. The astrocyte cultures were prepared as previously described and were obtained from either gender [7, 16]. Confluent cultures of human astrocytes were infected by incubation with viral stocks (20–50 ng p24/ml/1 × 10^6^ cells), HIV_ADA_ or HIV_JR-CSF_, using a previously described protocol [7, 8, 13].

### Cx30 siRNA

Three unique 29mer siRNA duplexes to human Cx30 were designed and obtained from Origene (Rockville, MD). siRNA (10 nM) transfection was performed with Oligofectamine (Invitrogen) according to the Origene application guide for Trilencer-29 siRNA. Minimal cell death was detected after transfection. Experiments were performed 2 days post transfection.

### Immunofluorescence

Astrocytes were grown on coverslips, fixed, and permeabilized in 70 % ethanol for 20 min at −20 °C. Cells were incubated in blocking solution for 30 min at room temperature and then in diluted primary antibody (anti-HIV-p24, anti-Cx43, anti-Cx30, and anti-GFAP; 1:50, 1:2000, 1:300, and 1:800, respectively) overnight at 4 °C. Cells were washed several times with PBS at room temperature and incubated with the appropriate secondary antibodies for at least 3 h at room temperature followed by another wash in PBS for 1 h. Cells were examined by confocal microscopy using an A1 confocal microscope (Nikon, Japan). Antibody specificity was confirmed by replacing the primary antibody with a non-specific myeloma protein of the same isotype or non-immune serum as we previously described [17, 18].

### Immunofluorescent and confocal microscopy analysis of human brain tissue samples

Postmortem human brain tissue sections from uninfected controls (four cases) and HIV-infected individuals with HIV encephalitis (HIVE, four cases) and minor cognitive disease (five cases) were analyzed by four color immunohistochemical staining for DAPI (nuclei staining), HIV-p24 (HIV viral protein), Cx43 or Cx30, and GFAP (an astrocyte marker). Sections of 10 μm were deparaffinized, underwent antigen retrieval, and blocked (5 mM EDTA, 1 % fish gelatin, 1 % essentially Ig-free BSA, 2 % human serum, and 2 % horse serum) for 60 min at room temperature and then incubated with anti-HIV-p24 (HIV viral protein), Cxs, and GFAP (an astrocyte marker) overnight at 4 °C. The sections were washed with PBS, incubated with secondary antibodies for 1 h at room temperature, followed by serial washes in PBS for 1 h. The samples were then mounted using Prolong Gold anti-fade reagent (Invitrogen) and examined by confocal microscopy. Specificity was confirmed by replacing the primary antibody with the appropriate isotype-matched control reagent, anti-IgG2A, or the IgG fraction of normal rabbit serum (Santa Cruz, Biotechnology).

### Western blot

Relative levels of Cx43, Cx30, and tubulin were determined by immunoblot as described [13, 19]. Astrocyte cultures were treated with HIV- tat or vehicle and harvested in Tris buffer, 10 mM pH 7.4, containing protease and phosphatase inhibitors (20 mM; pyrophosphate, 20 mM; NaF, 100 mM; NaVO_3_, 200 μM; leupeptin, 500 μg/ml; aprotinin, 40 μg/ml; soybean trypsin inhibitor, 2 mg/ml; benzamidine, 1 mg/ml; ω-amino caproic acid, 1 mg/ml; PMSF, 3 mM; and EDTA, 20 mM) [13, 19]. Cells were lysed, and the protein content of each cell lysate was determined using Bradford’s method [20] (Bio-Rad labs, Hercules, CA). Samples containing 20 μg of protein were used to analyze Cx43, Cx30, and tubulin. Proteins were separated in 7.5 % SDS-PAGE and electrophoretically transferred to nitrocellulose, which was then incubated sequentially with blocking solution (5 % non-fat milk in Tris-buffered saline); affinity-purified rabbit polyclonal antibodies prepared against Cx43, Cx30, and tubulin (1:2000, 1:500, and 1:2000, respectively); and anti-rabbit IgG conjugated to HRP. Antigen-antibody complexes were detected by ECL (Perkin Elmer, Boston, MA) and the resulting immunoblot signals were scanned, and densitometric analysis was performed using NIH-image software. All results were normalized to the values obtained for control conditions.

### Dye coupling

To evaluate the function of gap junction channels, the intercellular transfer of Lucifer Yellow (LY) (5 % *w*/*v* in 150 mM LiCl) was evaluated by microinjecting the dye into a single cell and evaluating the diffusion of the dye into neighboring cells, as previously described [21]. Cells were scored as coupled if dye transfer occurred to one or more adjacent cells. Dye transfer was evaluated using a fully motorized Zeiss Z1 microscope. Four independent experiments were performed in which a minimum of 20 cells were microinjected per experiment. The incidence and index of dye coupling was scored as the percentage of injections that resulted in dye transfer and the numbers of cells coupled to a single microinjected cell.

### Chromatin immunoprecipitation

Primary human astrocytes were transfected with pcDNA3.1+/tat101-flag (NIH repository, 10453) with Lipofectamine 2000. Twenty-four hours post transfection, chromatin immunoprecipitation (ChIP) analysis was performed for Cx43 and Cx30. In brief, cells were fixed and cross-linked using 1 % formaldehyde and washed in PBS, as we described with minor modifications [22]. Cells were homogenized, sonicated until 500-bp fragments were obtained using 20 pulses of 30 s on and 30 s off in a Microtip (Misonix, Inc, Microson XL-2000). Samples were pre-cleared with protein A/G and immunoprecipitated using anti-FLAG antibodies (Sigma). Specific binding of HIV-tat to Cx43 and Cx30 promoters was analyzed by qRT-PCR using a StepOnePlus Real Time PCR system (Life Technologies) and absolute blue QPCR SYBR low Rox Mix (Life Technologies). Single product amplification was confirmed by melting curve analysis, and primer efficacy was near or close to 100 % in all experiments. The human Cx43 promoter forward primer 5′-CCT CCT CCC AGT TGA GTC AG-3′ and reverse primer 5′-ACG CCA AGT GAT TGA ACT CC-3′ were described previously [23]. Human Cx30 promoter was analyzed using the following primers: forward 5′-TCC TGC ACT CCT TGC TCC TCA-3′ and reverse 5′-TCC CAC CTG CTG CGC CTT T [24].

### HIV-p24 ELISA

HIV-p24 concentrations in the medium of uninfected and HIV-infected cultures were determined by ELISA using a commercial kit from Perkin Elmer (Boston, MA).

### Statistical analysis

Mean differences were tested by non-parametric Kruskal-Wallis analysis or student’s *t* test. If a significant *F* value was obtained, means were compared with Bonferroni-Dunn multiple comparison test. A value of *p* < 0.05 was considered significant.

## Results

### Cx43, but not Cx30, is upregulated in human brain tissue sections obtained from subjects with HIV mild cognitive disease and encephalitis

**Fig. 1:**
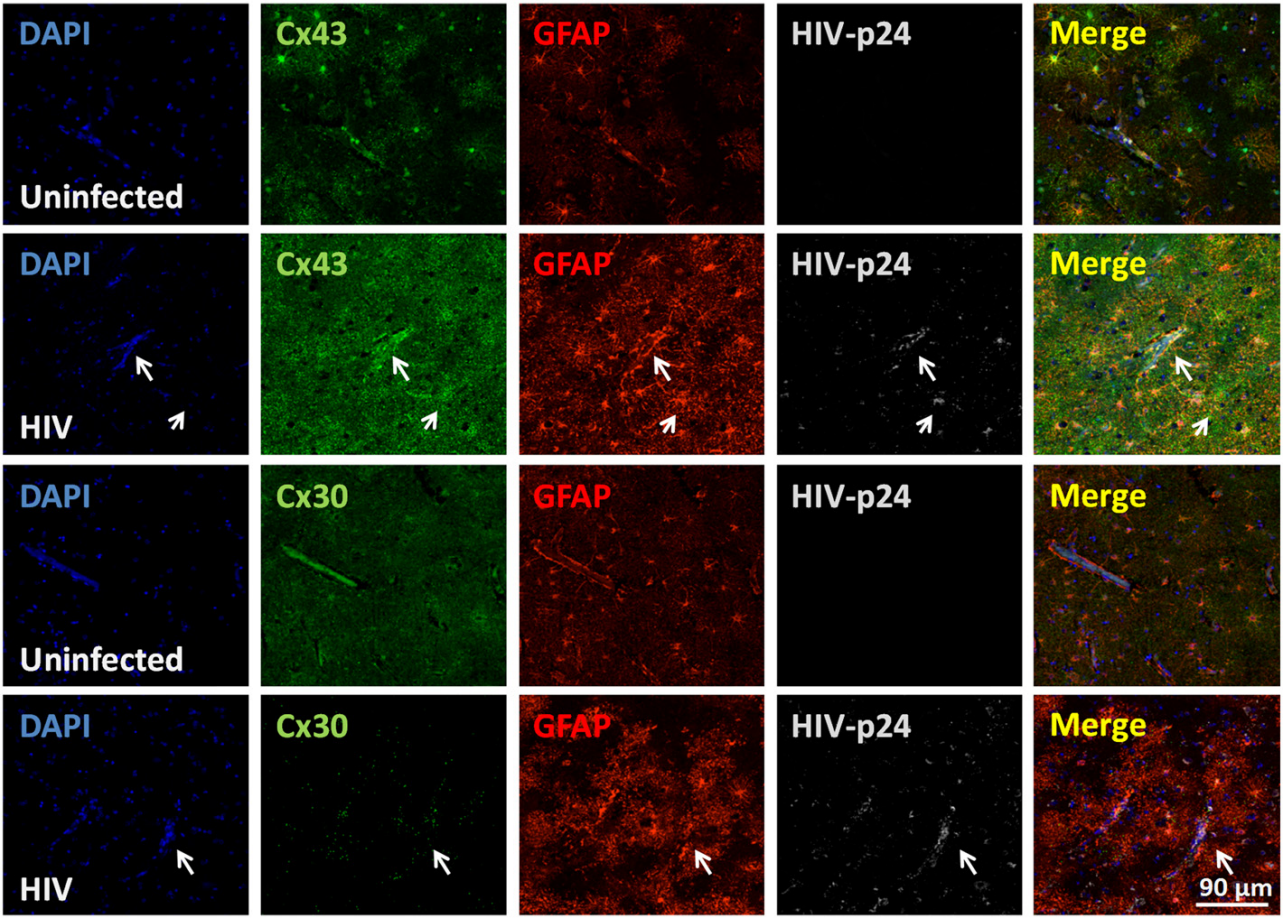
Glial expression of Cx43 is upregulated in HIV-infected individuals. Cx43 and Cx30 glial expression was evaluated using human brain tissue sections obtained from uninfected and HIV-infected individuals with mild and HIVE. Brain sections were evaluated by immunohistochemistry and confocal microscopy. Astrocyte expression of Cx43 or Cx30 (FITC *green*) was evaluated using glial fibrillary acid protein (GFAP, an astrocyte marker, *red staining*) and HIV infection by staining with HIV-p24 antibodies. In uninfected tissue sections, Cx43 and Cx30 localized in astrocytes (*uninfected row*). In contrast, in tissues obtained from HIV-infected individuals, Cx43 was highly upregulated, while Cx30 was downregulated. DAPI staining was used in counter staining. *Arrows* represent colocalization of GFAP, Cx43, and HIV-p24. Thus, HIV infection increases Cx43 expression, but not Cx30, in astrocytes

To examine the expression levels of astrocytic connexins, Cx43 and Cx30, we analyzed by immunofluorescence and subsequent confocal microscopy brain tissue section obtained from uninfected and HIV-infected subjects with cognitive impairment from the NNTC. Sections obtained from adult uninfected individuals showed Cx43 and Cx30 mainly localized in GFAP positive astrocytes (Fig. 1, uninfected). Confocal analysis of brain sections obtained from individuals with HIV cognitive impairment and HIV encephalitis (HIVE) showed increased expression of Cx43 in all astrocytes, especially the one positive for HIV-p24 proteins (Fig. 1, see arrows). In contrast, Cx30 in HIV-infected individuals was reduced (Fig. 1). No differences in Cx43 increased levels were detected between mild cognitive and HIVE (data not shown). In addition, GFAP expression increased and astrocytes become more positive, suggesting the presence of hypertrophic astrocytes (Fig. 1, GFAP, HIV). As we reported previously, the percentage of HIV-p24 positive astrocytes was 5.1 ± 2.15 % [7, 8, 25]. Negative controls using IgGs and control sera did not show positive staining (data not shown). Thus, HIV infection increased Cx43 expression in all HIV individuals analyzed.

### HIV-tat protein, but not other HIV proteins, increases Cx43 expression

Our previous data and the data described above indicate that HIV-infected astrocytes and surrounding cells have increased expression of Cx43 and gap junctions remain functional during HIV infection of the CNS [7, 8] and that HIV infection resulted in the opening of Cx43-containing hemichannels [26]. These results indicate that despite HIV infection and associated inflammation that normally result in gap junction closure, gap junctions and hemichannels are present and active. In other inflammatory and viral diseases, these channels are shut down [4, 5]. Thus, HIV infection is different. However, the mechanism(s) by which HIV regulates Cx expression in human astrocytes were unknown.

To characterize the contribution of specific HIV proteins to the increased Cx43 expression and maintenance of gap junction communication, we treated uninfected astrocyte cultures with the HIV (ADA or JR-CSF, R5 strains) for 7, 14, 21, and 28 days or with HIV recombinant proteins Vif (1, 100, and 300 ng/ml), Gag (p55, p24, or p7; 1, 100, and 300 ng/ml), Rev (1, 100, and 250 ng/ml), Nef (1, 100, and 250 ng/ml), gp120 (HIV_Bal_, HIV_JR-CSF_, or HIV_SF162_; 1, 50, and 100 nM), or HIV-tat_1–72_ (1, 100, and 300 ng/ml) for 6, 12, 24, and 48 h, and total expression of Cx43 (Table 1, data from all three concentrations were combined) and Cx30 (Table 2) were evaluated by western blot as a screening method. As a positive control for inflammation and decreased Cx43 expression, we used human astrocytes treated with IL-1β (10 ng/ml) as previously described [27].

**Table 1:** Expression of Cx43 by astrocytes after different HIV treatments

Cx43 expression in astrocytes (percentage of control, *n* = 5)				
Treatment (virus)	7 days	14 days	21 days	28 days
HIV_ADA_ (20 ng/ml)	No change	No change	No change	No change
HIV_JR-CSF_ (20 ng/ml)	No change	No change	No change	No change
Treatment (recombinant proteins)	6 h	12 h	24 h	48 h
Vif (1, 100, and 300 ng/ml)	No change	No change	No change	No change
Gag (p55, p24, or p7: 1, 100, and 300 ng/ml)	No change	No change	No change	No change
Rev (1, 100, and 250 ng/ml)	No change	No change	No change	No change
Nef (1, 100, and 250 ng/ml)	No change	No change	No change	No change
Gp120 (1, 50, and 100 nM)	−8.6 ± 1.3*	−5.4 ± 3.2*	−4.1 ± 1.2*	−3.1 ± 0.5*
HIV-tat_1–72_ (1, 100, and 300 ng/ml)	45.1 ± 12.3*	21.3 ± 8*	21.1 ± 2.2*	25.1 ± 2.5*
IL-1β (10 ng/ml)	−10.2 ± 5.2*	−16 ± 6.89*	−61.3 ± 11*	−69.9 ± 12.6*

Although we used three different concentrations for each HIV protein, no significant differences were detected among them. Thus, we combined the data from the three different concentrations

*p ≤ 0.05 (*n* = 4)

**Table 2:** Expression of Cx30 on astrocytes after different HIV treatments

Cx30 expression in astrocytes (percentage of control, *n* = 3)				
Treatment (virus)	7 days	14 days	21 days	28 days
HIV_ADA_ (20 ng/ml)	−12.3 ± 9.9*	−22.5 ± 8.9*	−32 ± 9.88*	−38.9 ± 7.9*
HIV_JR-CSF_ (20 ng/ml)	−28.7 ± 6.8*	−32.7 ± 11*	−49.8 ± 14.6*	−65.5 ± 21.2*
Treatment (recombinant proteins)	6 h	12 h	24 h	48 h
Vif (1, 100, and 300 ng/ml)	−3.8 ± 1.06*	−5.99 ± 2.3*	−8.88 ± 4.5 *	−8.98 ± 5.6*
Gag (p55, p24, or p7: 1, 100, and 300 ng/ml)	No change	No change	No change	No change
Rev (1, 100, and 250 ng/ml)	No change	No change	No change	No change
Nef (1, 100, and 250 ng/ml)	−5.3 ± 3.2*	−8.6 ± 3.43*	−12.7 ± 6.6*	−14.9 ± 7.45*
Gp120 (1, 50, and 100 nM)	−3.44 ± 2.1*	−5.6 ± 5.4*	−8.88 ± 4.3*	−9.15 ± 3.3*
HIV-tat_1–72_ (1, 100, and 300 ng/ml)	−9.02 ± 3.3*	−10.5 ± 5.4*	−17.5 ± 3.9*	−28.1 ± 11.8*
IL-1β (10 ng/ml)	−21.7 ± 8.9*	−36.54 ± 9*	−51.3 ± 9.3*	−52.3 ± 21.5*

Although we used three different concentrations for each HIV protein, no significant differences were detected among them. Thus, we combined the data from the three different concentrations

*p ≤ 0.05 (*n* = 4)

**Fig. 2:**
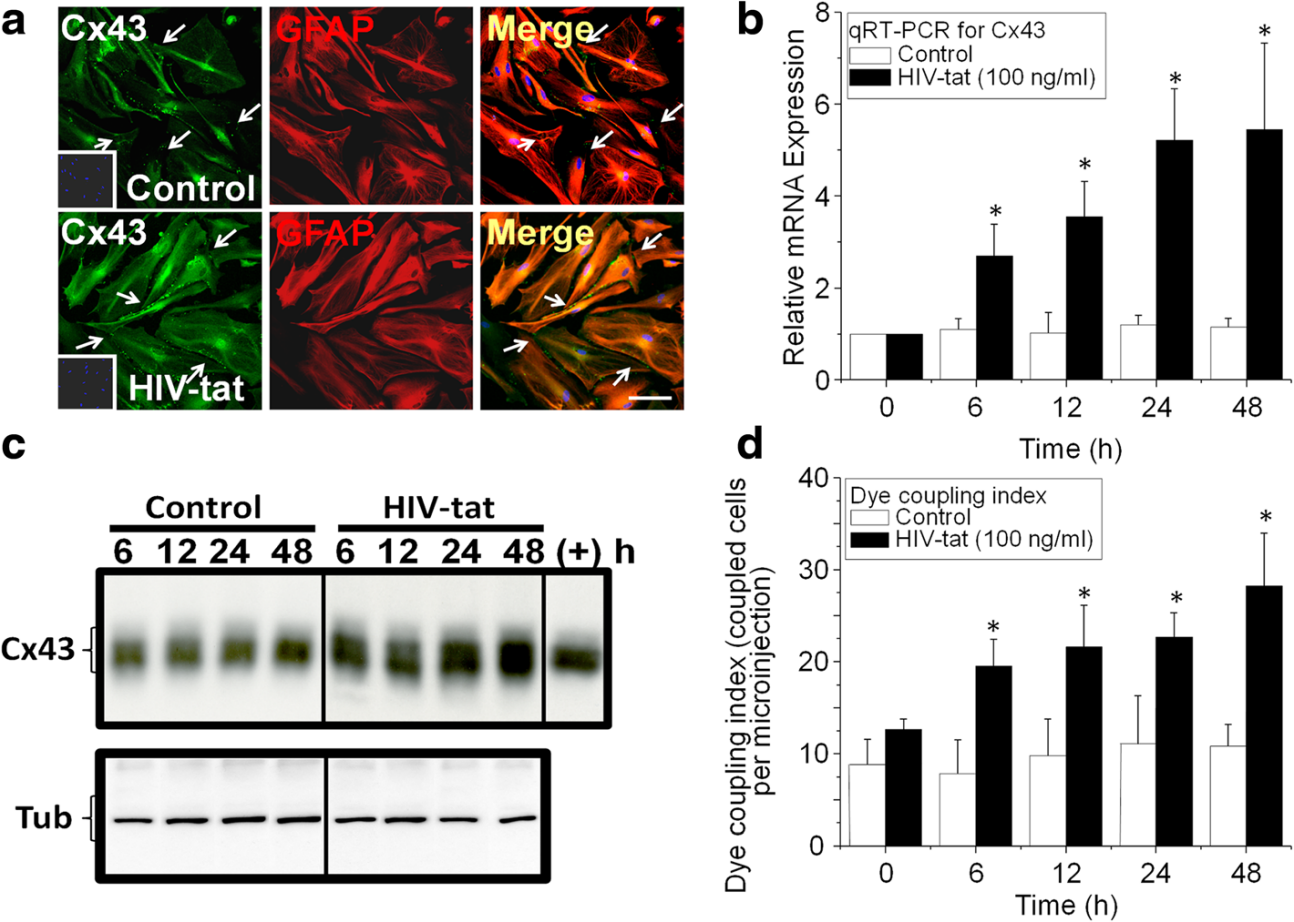
HIV-tat increased mRNA and Cx43 protein expression as well as gap junctional communication in human primary astrocytes. Human primary astrocytes were treated with recombinant HIV-tat protein (100 ng/ml), and expression and function of Cx43-containing channels were analyzed. **a** Staining for the nucleus (DAPI, *blue staining, in the insets in the left column*), Cx43 (Alexa 488, *green staining*), and GFAP (an astrocyte marker, Cy3, red *staining*) in untreated (control) and HIV-tat-treated conditions (HIV-tat) after 24 h of treatment. The *last panel* represents the merge of all colors. *Bar* 75 μm. *Arrows* denote gap junction plaques. **b** qRT-PCR for Cx43 and GAPDH mRNA using untreated (control) and HIV-tat-treated cultures of astrocytes at different time points (0, 6, 12, 24, and 48 h). No significant differences in mRNA Cx43 expression were detected in control cells (*white bars*). HIV-tat treatment increased Cx43 mRNA expression in a time-dependent manner (*p ≤ 0.001, *n* = 4, *black bars*). **c** Western blot analysis of Cx43 protein expression in control and HIV-tat-treated human astrocytes for 6, 12, 24, and 48 h. As a loading control, tubulin was used (tub). As a positive control for Cx43 and its phosphorylation, mouse astrocytes were used (+). **d** Dye coupling experiments using Lucifer Yellow (LY) showed that in both control and HIV-tat-treated cultures, dye spread into neighboring cells was 100 % (images not shown). However, HIV-tat increased the numbers of coupled astrocytes for each microinjection (*p ≤ 0.003, *n* = 4), indicating increased gap junctional communication

As we described, human astrocytes can be infected with both R5 and X4 strains of HIV [7, 8]. In agreement with other groups [10], low viral replication was detected 7, 14, 21, and 28 days post viral exposure to HIV_92UG021_ (X4), HIV_JR-CSF_ (R5), or HIV_ADA_ (R5) [7, 8]. HIV infected a small population of astrocytes (4.7 ± 2.8 %). Viral replication of all viral isolates tested in these cultures was maximal after 14 days post infection, as determined by HIV-p24 ELISA, and remained stable until 28 days (data not represented). Despite observing increased Cx43 in HIV-infected cells and surrounding cells (see [8]), no significant differences in Cx43 expression by western blot analysis was detected, probably due to the low numbers of HIV-infected cells as compared to the uninfected cells (see Table 1). The constancy of Cx43 expression in these cultures contrasts to the high expression seen in human brain tissue (Fig. 1), which shows increase in Cx43 immunolabling in uninfected as well as infected cells. The greater expression in the diseased brain is likely to be a result of the much longer exposure than in the cultured cells. The difference may be that secreted HIV-tat is acting on uninfected (Fig. 5). HIV-tat may be a toxic signal that increases Cx43 expression in uninfected cells and formation of Cx43 hemichannels that allow excessive influx of Ca^2+^ and efflux of essential metabolites. HIV-tat applied to astrocytes in culture clearly increases Cx43 expression (Fig. 2).

Furthermore, Cx43 protein expression was not altered by any concentration of recombinant vif, gag, rev, or nef at any time point examined (Table 1). However, all gp120 concentrations tested decreased Cx43 expression by 3 to 9 % (*p* < 0.005, *n* =4), suggesting that this HIV protein is not involved in maintaining or increasing Cx43 expression (Table 1). In contrast, exon 1 (HIV-tat_1–72_) or both exons (HIV-tat_1–101_; data not shown) of HIV-tat resulted in upregulation of Cx43 protein expression at all concentrations and time points tested (Table 1).

In contrast, HIV infection (ADA and JR-CSF) and the recombinant proteins vif, nef, and gp120 decreased Cx30 protein expression at all concentrations tested (Table 2). Gag and Rev did not alter Cx30 protein expression (Table 2). HIV-tat resulted in decreased expression of Cx30 at all three concentrations and times tested (Table 2). These results indicate that HIV-tat contributes to the maintained or increased expression of Cx43 but contributes to the reduction in Cx30 expression.

### HIV-tat upregulates Cx43 mRNA and protein as well as increases gap junctional communication

To determine the mechanism(s) by which HIV-tat increased Cx43 protein expression, we evaluated Cx43 mRNA and protein, as well as gap junctional communication after HIV-tat treatment. Human astrocyte cultures were treated with 1, 100, or 300 ng/ml of HIV-tat for 6, 12, 24, and 48 h, and cells were analyzed by confocal microscopy, qRT-PCR, western blotting, and dye coupling (Fig. 2, only 100 ng/ml of HIV-tat is shown).

Cx43, GFAP, and nuclear staining, and subsequent analysis by confocal microscopy, demonstrated that Cx43 is mostly located in the plasma membrane and the cytoplasm in control conditions as expected (Fig. 2a, control). When cells were treated with HIV-tat, Cx43 staining in the membrane and cytoplasm was increased in all astrocytes (Fig. 2a, HIV-tat, 24 h). qRT-PCR analysis for Cx43 and GAPDH mRNA demonstrated that HIV-tat upregulates Cx43 mRNA up to ~sixfold as compared to untreated cells (Fig. 2b, *p ≤ 0.002 at all-time points analyzed). Western blot analysis of these samples indicated that HIV-tat treatment increased Cx43 protein 6 to 48 h post treatment (Fig. 2c at all-time points analyzed, *p* ≤ 2.3 × 10^−5^). Tubulin was used as a loading control (Fig. 2c, tub).

To examine the function of these channels, dye coupling was performed. In control conditions, astrocytes were 100 % coupled to other surrounding astrocytes (data not shown) with an average of 10 ± 2.6 cells coupled for each cell microinjected with LY (Fig. 2d, dye coupling index). HIV-tat treatment of human astrocytes increased the numbers of coupled cells up to 27.3 ± 4.76 cells per microinjection (Fig. 2d, *p < 0.003), suggesting that toxic signals generated in a few HIV-infected astrocytes could reach longer distances by a HIV-tat-dependent mechanism.

Furthermore, HIV-tat effects on Cx43 expression and gap junctional communication were specific to human cells, because treatment of mouse astrocytes with HIV-tat (100 ng/ml) for 6 to 72 h did not alter Cx43 expression or gap junctional communication, suggesting that the effect is species specific, as is HIV infectivity (data not shown).

### HIV-tat protein downregulates Cx30 expression, while Cx30 does not contribute to the increased coupling index observed after HIV-tat treatment

**Fig. 3:**
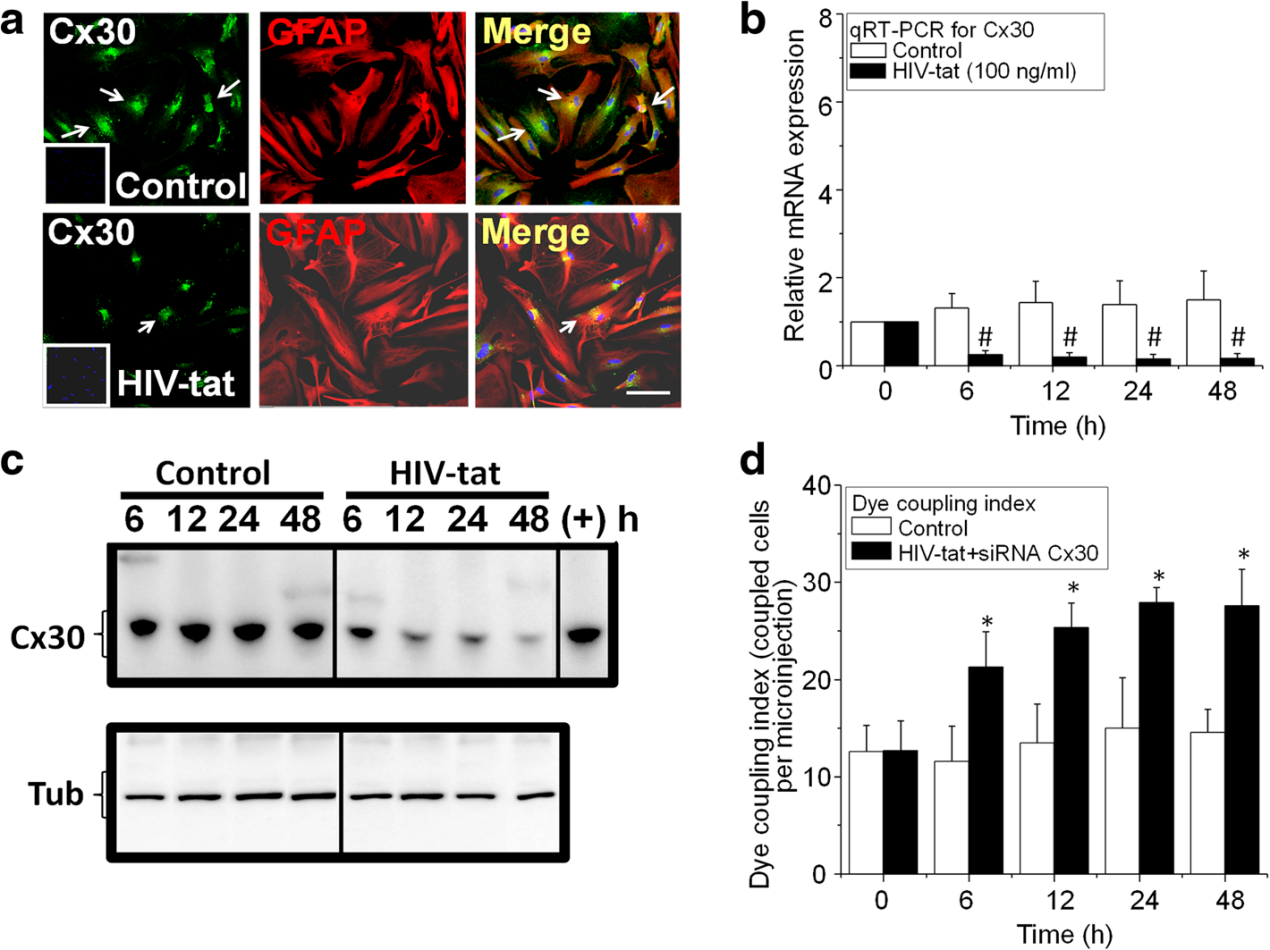
HIV-tat did not increase Cx30 and mRNA protein expression and did not contribute to the enhanced gap junctional communication induced by HIV-tat in human primary astrocytes. Human primary astrocytes were treated with recombinant HIV-tat protein (100 ng/ml), and expression and function of Cx30-containing channels were analyzed. **a** Labeling for nuclei (DAPI, *blue staining*), Cx30 (Alexa 488, *green staining*), and GFAP (Cy3, *red staining*) in untreated conditions (control) and after 24 h HIV-tat treatment. The *last panel* represents the merge of all colors. *Bar* 75 μm. **b** qRT-PCR for Cx30 and GAPDH using untreated and HIV-tat-treated (24 h) cultures of astrocytes. No significant differences in mRNA Cx30 expression were detected in control cells (*white bars*). HIV-tat treatment decreased Cx30 mRNA expression in a time-dependent manner (^#^ *p* ≤ 0.001, *n* = 4, *black bars*). **c** Western blot analysis of Cx30 protein expression in control and HIV-tat-treated human astrocytes. Tubulin (tub) was used as a loading control. As a Cx30 positive control, mouse brain was used (+). HIV-tat decreased Cx30 expression in a time-dependent manner (Fig. 2c). **d** To examine the contribution of Cx30 to the increased gap junctional communication induced by HIV-tat, we reduced further the expression of Cx30 using siRNA. In this condition, we reduced Cx30 by at least 80 %; however, no changes in dye coupling were observed, suggesting that Cx43 mediate most of the communication (*n* = 4, *p* ≤ 0.005)

To determine whether HIV-tat also regulates expression of Cx30, human primary cultures of astrocytes were treated with HIV-tat (1, 100, or 300 ng/ml), and expression and localization of Cx30 was examined as described for Cx43. Our data indicate that HIV-tat downregulates Cx30 in human astrocytes as demonstrated by immunofluorescence (Fig. 3a), qRT-PCR (Fig. 3b), and western blotting (Fig. 3c). Despite the downregulation of Cx30 by HIV-tat, protein expression is significant. Thus, to determine whether the low levels of Cx30 after HIV-tat treatment contribute to the increased gap junctional communication induced by HIV-tat , Cx30 siRNA was transfected into primary cultures of human astrocytes using Lipofectamine 2000, and gap junctional communication was determined as described above. Cx30 siRNA reduced Cx30 protein expression in 78.7 ± 12.65 % instead of 9 to 28 % observed with just HIV-tat (Fig. 3 and Table 2). Despite the reduction of Cx30 protein by the combination of HIV-tat and Cx30 siRNA treatment (Fig. 3d), we still detected a significant increase in coupling index, indicating that HIV-tat mainly targets Cx43 and that increased gap junctional communication in response to HIV-tat treatment is mediated by Cx43-containing channels.

### HIV-tat binds to the Cx43 promoter, but not to the Cx30 promoter

The main function of HIV-tat protein in the HIV cell cycle is to enhance and stabilize Pol II transcription of the viral DNA. HIV-tat binds to the RNA stem-loop structure, the trans-activating response (TAR) element, found at the 5′ ends of nascent HIV transcripts. HIV-tat’s binding to TAR alters the formation of the transcription complex and recruits the transcription factor P-TEFb of CDK9 and cyclin T1, to increase the production of full length viral RNA [28, 29]. However, it was unknown whether HIV-tat binds and affects Cx43 promoter activity.

**Fig. 4:**
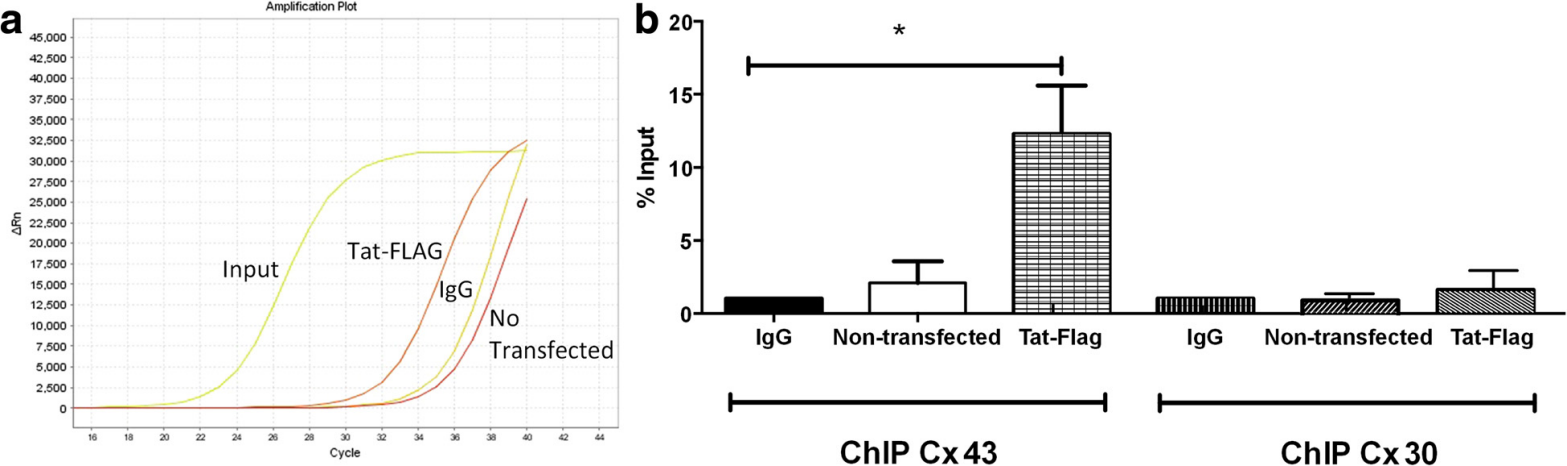
HIV-tat binds to the Cx43 promoter, but not to the Cx30 promoter, in human primary astrocytes. Human primary astrocytes were transfected with pcDNA3.1+/tat101-flag, and binding of this HIV protein to the Cx43 and Cx30 promoter was assayed by ChIP. **a** Representative PCR curves of Cx43 promoter DNA amplification after 24 h of HIV-tat-FLAG transfection and subsequent ChIP. In the plot, amplification of input, tat-FLAG, irrelevant IgG, and non-transfected are shown. **b** Compilation of four ChIP experiments. Primary astrocytes were transfected with HIV-tat-FLAG to examine its binding to the Cx43 promoter using ChIP. Negative controls for the ChIP using IgG, or non-transfected astrocytes (data not shown) did not show significant amplification. Only HIV-tat-FLAG amplify and increase the binding at least 15-fold as compared to the input amplification (B, ChIP Cx30). ChIP analysis for Cx30 like that for Cx43 showed no HIV-tat-FLAG binding to the Cx30 promoter (*n* = 4, *p* = 0.004)

Human primary astrocytes were transfected with HIV-tat-FLAG for 24 h, and ChIP analysis was performed to demonstrate binding of HIV-tat-FLAG to the Cx43 promoter as shown in Fig. 4a, b (*n* = 4). HIV-tat-FLAG immunoprecipitation pulls down the Cx43 promoter (see amplification curves of Cx43 DNA, tat-FLAG), demonstrating that HIV-tat-FLAG binds to this promoter (Fig. 4b, ChIP Cx43 tat-FLAG). No binding of HIV-tat-FLAG to the Cx43 promoter was detected with any negative controls, including an irrelevant isotype-IgG matched, or untransfected cells (Fig. 4b, IgG and non-transfected). In agreement with our data that HIV-tat did not increase Cx30 expression, ChIP analysis of the Cx30 promoter did not show any binding (Fig. 4b, ChIP Cx30). These data indicate that HIV-tat increases expression of Cx43, but not Cx30, by a mechanism that involves increased HIV-tat’s binding to the Cx43 promoter, Cx43 mRNA production, protein synthesis, and gap junctional communication. We propose that HIV-tat is an early viral factor and enables HIV-infected cells to maintain Cx43 expression, resulting in increased gap junctional communication between the few HIV-infected astrocytes and the surrounding uninfected cells, enabling spread of toxic signals.

## Discussion

In this report, we demonstrate that HIV-tat, the transactivator of the virus, binds to the Cx43 promoter and increases Cx43 mRNA, protein, and gap junctional communication. Thus, HIV-tat not only regulates HIV transcription but also regulates an important host gene within the CNS. Our published data demonstrate that the maintenance or increase of Cx43 and gap junctional communication as well as increased hemichannel opening are essential to mediate bystander apoptosis between the few infected astrocytes (4.7 ± 2.8 % in vitro and 8.2 ± 3.9 % in vivo using an SIV model) and neighboring uninfected cells such as uninfected astrocytes, neurons, and brain endothelial cells [5, 7, 8, 25]. Our previous studies also demonstrated that maintenance of gap junctional communication by HIV infection enables intracellular toxic signals, including cytochrome C-related signals (cytochrome C cannot cross gap junctions due to its size), IP_3_, and calcium generated in a few HIV-infected astrocytes to spread to uninfected communicated cells resulting in amplification of apoptosis and inflammation. All of these changes are independent of active HIV replication. Thus, bystander damage is associated with infection, but not with replication, as HIV-tat is produced by HIV-infected cells even in the presence of anti-retrovirals [30, 31]. This may be an important mechanism mediating HIV CNS damage despite inefficient HIV-infected cells or replication.

Our data reflect the current status of the majority of people infected with HIV, where HIV replication is minimal due to successful antiretroviral treatment. However, despite low replication and normal CD4 counts, 50–70 % of the HIV-infected population shows some signs of cognitive impairment [32, 33]. Thus, it is not the virus mediating these effects; it is more likely that these are amplification systems altered by viral factors that are not impacted by antiretroviral treatment. Currently, antiretroviral treatments have no effect on HIV-tat production. Thus, this protein is produced and secreted independently of effective viral replication [31], suggesting that the effects observed in HIV-infected astrocytes could be present in HIV-infected individuals regardless of successful combination antiretroviral therapy (cART). In addition to the effects of HIV-tat on gap junctional communication, this HIV protein is also directly neurotoxic, capable of triggering inflammation and altering several synaptic components [13, 34, 35].

Our data and that of others have demonstrated a key role of astrocytes in the pathogenesis of NeuroAIDS by mechanisms that involve gap junctional communication, hemichannels, and Wnt pathways [11, 25, 36–39]. Thus, we propose that HIV-tat upregulates Cx-containing channels, including gap junctions and hemichannels, to spread toxic signals into neighboring cells by increasing the radius of diffusion of intracellular factors through gap junctions.

Normally, gap junctions are master regulators of electrical and metabolic coordination in most tissues including the CNS. It has recently become evident that connexin-containing channels, gap junctions and hemichannels, are also key regulators of learning and memory, long-term potentiation, and depression; the ratio of NR2a/NR2b; and electrical oscillations [40–45]. Thus, any changes in gap junctional communication or Cx expression will affect CNS function, as observed in HIV-infected individuals. These channels in parenchymal cells are shut down in response to inflammatory stimuli such as cytokines, chemokines, and pathogens [4, 5, 46]. However, HIV infection of astrocytes has a different effect, in that connexin-containing channels are maintained or increased, resulting in bystander apoptosis of uninfected cells. In agreement, some reports indicate that functional gap junction channels may help to amplify ischemic damage by enhancing the propagation of pro-apoptotic death signals between dying and healthy cells [47]. Additionally, α-particle-irradiated cells also transmit pro-apoptotic signals through gap junctions to non-irradiated cells [48]. These results suggest that gap junction channels can be used to spread damage into healthy areas.

Direct binding of HIV-tat to the Cx43 promoter was unexpected. The promoters for Cxs and long-term repeats (LTR) of HIV are very different and share the use of few transcription factors. The human Cx43 promoter contains several putative transcription factor binding sites including AP-1, AP-2, cAMP response elements, Ets, specificity protein-1 consensus sites, several sequences resembling half the palindromic estrogen response elements and progesterone response elements, and an activator and a repressor site that have been functionally characterized in the mouse gene [23, 49, 50]. There are no current elements shared by the virus and the Cx43 promoter that enable binding of Pol II in similar sites. HIV transcription requires the transcription factors TFIID, SP1, NF-kB, NFAT, and AP-1, and upstream also contains USF, Ets, and LEF-1 binding sites [51]. Interestingly, we did not observe that HIV-tat increased Cx43 expression in mouse astrocytes, suggesting that HIV-tat effects are human DNA specific, similar to HIV infection. This may be due to the expression of cyclin T1, a human-specific protein. To characterize these differences, further studies are required to identify the areas of binding of Pol II to the Cx43 promoter, transcription factors, and how HIV-tat regulates these interactions.

Furthermore, HIV-tat binding to the Cx43 promoter was specific, because no binding was detected to the Cx30 promoter. Currently, there are few reports describing the sequences and binding sites in this promoter. It is known that Cx30 transcription can start in different exons, for example, in the epidermis, transcription begins in exon 1, but in the CNS, it starts in exon 3. In the promoter region of the Cx30 human gene, upstream of exon 1, a TATA motif, several potential binding sites for sp1, and a consensus sequence for early growth response gene products (Egr)-binding are present [24]. However, any similarities or differences in the promoter need further description to identify the differences in HIV-tat sensitivity.

Our results suggest that blocking secretion or production of HIV-tat within the CNS or reducing gap junctional communication to physiological levels is a novel potential therapeutic approach to reduce the ongoing CNS compromise observed in a large population of HIV-infected individuals.

## Conclusions

**Fig. 5:**
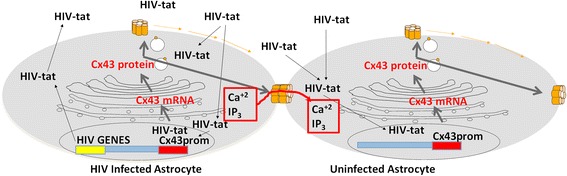
Schematic representation of our working model. We believe HIV infects a small population of astrocytes, inducing the expression of HIV-tat and subsequent upregulation of expression of Cx43. This upregulation of Cx43 expression results in the maintenance of gap junctional communication and opening of Cx43 hemichannels on the surface of the astrocytes. Both Cx43-containing channels, GJ and hemichannels, enable toxic intracellular signals (probably IP_3_ and calcium related) to spread into uninfected neighboring cells resulting in apoptosis. In contrast, HIV-infected astrocytes survive apoptosis, generating CNS reservoirs

The main conclusion of our work is that HIV-tat upregulates expression of Cx43 and maintains functional gap junctional communication by direct binding or interaction to the Connexin43 promoter. This upregulation of Cx43-containing channels allows toxic signals generated in few HIV-infected astrocytes to spread into uninfected cells by a gap junction or hemichannel- dependent mechanism resulting in bystander apoptosis of CNS cells (see summary in Fig. 5).
